# *NLRP7* in Hydatidiform Mole and Gestational Trophoblastic Neoplasia: From Maternal-Effect Biology to a Genetic Permissiveness Framework for Integrated Cancer Surveillance and Reproductive Decision-Making

**DOI:** 10.3390/cancers18152386

**Published:** 2026-07-24

**Authors:** Wei Xue, Tianyi Chen, Yang Xiang, Junjun Yang

**Affiliations:** National Clinical Research Center for Women’s Health and Obstetric and Gynecologic Diseases, Department of Obstetrics and Gynecology, Peking Union Medical College Hospital, Chinese Academy of Medical Sciences & Peking Union Medical College, Beijing 100730, China; xuewei@pumch.cn (W.X.); s2024001130@student.pumc.edu.cn (T.C.); xiangy@pumch.cn (Y.X.)

**Keywords:** *NLRP7*, hydatidiform mole, gestational trophoblastic neoplasia, choriocarcinoma, maternal-effect gene, genomic imprinting, subcortical maternal complex, assisted reproduction, preimplantation genetic testing, cancer predisposition

## Abstract

**Background/Objectives**: Biallelic mutations of *NLRP7*, a maternal-effect gene encoding a key component of the subcortical maternal complex, are the major monogenic cause of recurrent hydatidiform mole (RHM) and confer a substantial lifetime risk of gestational trophoblastic neoplasia (GTN), including choriocarcinoma. Although oocyte donation is currently the standard reproductive option, anecdotal live births from autologous oocytes in carriers of specific missense variants raise mechanistic and clinical questions that have not been integrated. We aimed to synthesize the current understanding of *NLRP7* biology, oncogenic mechanisms in GTN, and genotype-stratified reproductive outcomes, and to propose a genetic permissiveness framework that links cancer surveillance with reproductive decision-making. We also place this monogenic disease in an epidemiological context, given the marked geographical variation in molar pregnancy incidence. **Methods**: PubMed, Embase and Web of Science were searched from January 2006 to December 2025 using terms related to *NLRP7*, hydatidiform mole, GTN, choriocarcinoma, maternal-effect genes, genomic imprinting, and assisted reproduction. Original studies, case series, expert consensus statements, and reviews were included. **Results**: *NLRP7* loss disrupts subcortical maternal complex assembly, maternal-DMR methylation, trophoblast differentiation, and HLA-C-dependent immune privilege; in choriocarcinoma, *NLRP7* is conversely reported to be up-regulated, where it has been implicated in tumor proliferation and immune evasion through inflammasome-independent pathways. Genetic permissiveness for autologous-oocyte pregnancy is mutation-type dependent: complete loss-of-function variants confer near-zero permissiveness, whereas missense variants with retained partial function may permit rare normal pregnancies. **Conclusions**: A genetic-permissiveness framework integrating variant-level functional data, GTN risk, and reproductive priorities can support shared decision-making in this rare but high-stakes clinical scenario, and it identifies clear directions for future translational research. We emphasize, however, that this framework has not been prospectively validated and should be regarded as hypothesis-generating rather than as a clinically established decision-making model. Recognizing the biallelic *NLRP7* mutation as a heritable cancer-predisposition state further supports genotype-informed, risk-stratified oncological surveillance.

## Simple Summary

A small number of women lose every pregnancy to a hydatidiform mole, an abnormal pregnancy that can transform into a malignant tumor called gestational trophoblastic neoplasia. In many of these women, the cause is a defect in both copies of the *NLRP7* gene, which controls how very early embryos use the mother’s genetic instructions. Because the egg itself carries the defect, the standard advice is to use donated eggs. However, very rare patients with certain “milder” *NLRP7* changes have given birth to healthy children using their own eggs, and the molecular reasons remain unclear. We review what *NLRP7* does in normal pregnancy, how its loss drives both molar disease and its progression to cancer, and why some patients may retain a narrow biological window for pregnancy with their own eggs. We then propose a “genetic permissiveness” framework to support individualized counseling and to bridge cancer surveillance with reproductive planning.

## 1. Introduction

Hydatidiform mole (HM) is a pre-malignant gestational lesion characterized by hydropic chorionic villi and aberrant trophoblast proliferation. It is the most common form of gestational trophoblastic disease (GTD), and approximately 15–20% of complete moles progress to gestational trophoblastic neoplasia (GTN), which encompasses invasive mole, choriocarcinoma, placental-site trophoblastic tumor and epithelioid trophoblastic tumor [1,2,3]. The reported incidence of HM displays marked geographical variation [4,5], ranging from roughly 0.5 to 1 per 1000 pregnancies in North America and Europe to several-fold higher rates in parts of South-East Asia, Latin America and the Middle East; this heterogeneity reflects a combination of true differences in risk factors (maternal age extremes [6], prior molar pregnancy, nutritional and possibly consanguinity-related factors) and differences in case ascertainment and pathological classification. More recent population-based and contemporary-cohort data [7] are consistent with this range, reporting roughly 1–2 per 1000 pregnancies in Europe and North America against markedly higher rates—up to about 10 per 1000—in parts of South and South-East Asia, with a recent large Chinese single-center cohort likewise documenting substantial GTN progression among molar pregnancies [1,8]. Such epidemiological gradients are clinically consequential because they shape the pre-test probability of recurrent and genetically determined disease and therefore the threshold for genetic investigation in any given population. Although most HM cases are sporadic and androgenetic, a small subset of women experiences recurrent HM (RHM), with extreme phenotypes of seven or more consecutive molar pregnancies described in the literature [9,10,11]. RHM imposes both a profound reproductive-psychological burden and a markedly elevated oncological risk on affected women [12].

The identification of biallelic *NLRP7* (NLR family pyrin domain-containing 7) mutations as the principal genetic cause of familial RHM was a landmark discovery [13,14,15,16,17,18]. *NLRP7* is a maternal-effect gene whose protein product accumulates during oogenesis, participates in the subcortical maternal complex (SCMC), and is essential for maternal-DMR methylation and the orderly progression of the early embryo through zygotic genome activation (ZGA) [19,20]. The widespread adoption of next-generation sequencing has uncovered a growing spectrum of pathogenic *NLRP7* variants, including in patients diagnosed only after repeated assisted-reproduction failures [21], and methylation-array-based diagnostic strategies are emerging as adjunctive tools [22].

Beyond reproductive failure, *NLRP7*-driven RHM carries significant oncological implications. In several cohort studies, women with biallelic *NLRP7* mutations have shown a higher cumulative incidence of GTN than the sporadic-HM population, with cases of multi-agent-chemotherapy-requiring high-risk GTN refractory to single-agent regimens [23,24]; precise quantification of this excess risk is, however, limited by the small size and ascertainment bias of available cohorts. Detection of biparental diploidy in molar tissue—rather than the expected androgenetic pattern—should therefore prompt early genetic testing for maternal-effect-gene defects, with direct implications for both cancer surveillance and counseling on subsequent pregnancies [25]. Mechanistically, choriocarcinoma cells paradoxically up-regulate *NLRP7* protein, which sustains tumor proliferation, three-dimensional reorganization, and an immunosuppressive microenvironment through inflammasome-independent pathways [26,27].

Against this background, a central—and largely unanswered—clinical question persists: can a woman with biallelic *NLRP7* mutations achieve a normal pregnancy using her own oocytes? Current mainstream practice favors oocyte donation because virtually every autologous-oocyte pregnancy in this population terminates as HM [28]. Nevertheless, rare and reproducible reports of live births from carriers of specific missense variants suggest that “genetic permissiveness” for autologous pregnancy may be mutation-type dependent [29]. The development of patient-derived induced pluripotent stem cell (iPSC) trophoblast differentiation models has begun to provide functional traction on this question [30].

In this review, we (i) summarize the maternal-effect biology of *NLRP7* and the molecular consequences of its loss in oocytes and trophoblasts; (ii) integrate the oncogenic mechanisms by which *NLRP7* contributes to malignant progression from HM to GTN/choriocarcinoma; (iii) appraise the epidemiology of *NLRP7*-associated disease, including the genetically determined fraction of RHM and the excess GTN risk it confers; (iv) collate genotype-stratified reproductive outcomes; and (v) propose a “genetic permissiveness” framework that explicitly bridges cancer surveillance and reproductive decision-making for these high-risk women. To our knowledge, this is among the first reviews to frame genetically determined RHM explicitly as a putative heritable cancer predisposition state and to integrate variant-level functional data into a single decision architecture that simultaneously addresses oncological surveillance and reproductive choice. We stress that the clinical evidence underpinning this characterization remains limited: the direct association of biallelic *NLRP7* mutation with GTN derives largely from single-center, small-sample observations [23,24], while the remaining support is mechanistic or experimental. The cancer predisposition framing should therefore be read as a biologically motivated hypothesis requiring confirmation in larger, population-based cohorts rather than as an established clinical classification. The framework is intended as a hypothesis-generating clinical counseling tool and not as a replacement for individualized expert care; we therefore emphasize its evidence base, limitations, and the empirical questions whose resolution would refine it.

## 2. Materials and Methods

A comprehensive, narrative-review literature search was performed in PubMed, Embase, and Web of Science for articles published between 1 January 2006 (the year *NLRP7* was first identified as a causative gene for RHM) and 31 December 2025. The search strategy combined (“*NLRP7*” OR “*NALP7*” OR “NLR family pyrin domain-containing 7”) with terms related to gestational trophoblastic disease (“hydatidiform mole” OR “molar pregnancy” OR “gestational trophoblastic neoplasia” OR “choriocarcinoma” OR “recurrent molar”), maternal-effect biology (“maternal effect gene” OR “subcortical maternal complex” OR “genomic imprinting”), tumor biology (“trophoblast” OR “trophoblastic tumor” OR “tumor microenvironment”), and reproductive medicine (“oocyte donation” OR “preimplantation genetic testing” OR “assisted reproduction”). Reference lists of key publications, recent expert consensus statements [12], and pre-print servers (medRxiv, bioRxiv) were hand-searched.

Inclusion criteria: (i) original research articles, case reports/series, and reviews addressing *NLRP7* gene function, mutation spectrum, genotype–phenotype correlations, trophoblast biology, GTN/choriocarcinoma mechanisms, or reproductive outcomes in *NLRP7* mutation carriers; (ii) studies reporting assisted-reproductive strategies for RHM patients. Exclusion criteria: (i) non-English publications without an English abstract; (ii) conference abstracts without subsequent full publication. After de-duplication of 156 records, 87 articles met preliminary screening; 40 articles were retained for the final synthesis after full-text review. Because this is a narrative review rather than a systematic review or meta-analysis, no formal risk-of-bias assessment was performed; however, priority was given to studies with larger sample sizes, functional validation in vitro or in iPSC models, multicenter design, or expert-consensus status. Where claims rest on small case series or single-family observations, this is explicitly stated. The review was conducted in accordance with the SANRA scale for the assessment of narrative-review articles.

## 3. NLRP7: Maternal-Effect Biology and Functions in the Oocyte–Embryo Transition

### 3.1. Gene Structure, Expression and Domain Architecture

*NLRP7* is located at chromosome 19q13.42, spans approximately 17 kb across 13 exons, and encodes a 1037-amino-acid protein. As a member of the NLRP subfamily of NOD-like receptors, *NLRP7* contains an N-terminal pyrin domain (PYD) mediating homotypic protein–protein interactions, a central NACHT nucleotide-binding-and-oligomerization domain capable of ATP-dependent oligomerization, and a C-terminal leucine-rich-repeat (LRR) domain involved in ligand recognition and autoinhibition.

*NLRP7* expression is strikingly cell-type-restricted. It is highly enriched in human ovarian oocytes and preimplantation embryos, with peak transcript abundance during cleavage stages, consistent with a critical role during the maternal-to-zygotic transition before ZGA [31]. Lower-level expression is detectable in trophoblasts, monocytes and several immune-cell lineages, reflecting pleiotropic function [32]. Notably, *NLRP7* has undergone evolutionary degeneration in rodents, so functional studies depend on primate or human cellular models [33].

### 3.2. The Subcortical Maternal Complex (SCMC)

The SCMC is a multi-protein assembly restricted to the subcortical region of mammalian oocytes and early embryos. In humans, the components identified to date comprise *NLRP5*, *NLRP2*, *NLRP7*, *KHDC3L*, *OOEP*, *TLE6*, *PADI6* and *ZBED3* [34,35,36,37,38,39], and these act as an indispensable scaffold during early embryonic development [40,41]. It should be noted that SCMC composition is not fully conserved across species: rodents carry a single *Nlrp2* gene that appears to combine functions of the human *NLRP2* and *NLRP7* paralogues, and the relative stoichiometry of core members differs between human and mouse; the composition discussed here therefore refers to the human complex. Co-immunoprecipitation studies demonstrate direct interactions between *NLRP7* and several SCMC core members (*NLRP5*, *TLE6*, *PADI6*, *KHDC3L*, *OOEP*), consistent with a structural role in complex integrity [42]. Loss of *NLRP7* function destabilizes SCMC assembly and triggers early-embryonic developmental arrest [43].

Recent cryo-electron-microscopy work has resolved the architecture of the murine SCMC core [44,45]: the NACHT and LRR domains of *MATER* (the mouse *NLRP5* homolog) wrap around *TLE6* and *FLOPED* to form the core scaffold, with two core complexes dimerizing via LRR–domain interactions. Clinically relevant variants disrupt this assembly, providing structural context for how human *NLRP7* mutations precipitate developmental failure. *NLRP7* also interacts with alternative-splicing factors (*DDX39B*, *PRPF8*) and the DNA-damage-repair protein *PARP1*, contributing to genomic stability during early human embryogenesis [46].

### 3.3. Maintenance of Maternal Genomic Imprinting

Genomic imprinting is the parent-of-origin-dependent monoallelic expression of a defined set of genes, governed by precisely placed differentially methylated regions (DMRs) [47,48]. As a maternal-effect gene, *NLRP7* supports maintenance of maternal-DMR methylation during oocyte growth and the immediate post-fertilization period through its participation in the SCMC [49,50]. Miscarriage tissues from biallelic *NLRP7* carriers exhibit multilocus imprinting disturbances, with widespread maternal-DMR hypomethylation at canonical loci such as *KCNQ1OT1*, *PEG3* and *SNRPN* [51].

Genome-wide methylation analyses extend this picture: more than 60 placenta-specific imprinted loci show maternal-methylation loss in biparental molar tissues, while paternally methylated loci (e.g., *H19*) remain unaffected. The HM phenotype therefore reflects systematic disruption of placenta-specific imprinting rather than isolated lesions at classical imprinted loci. *NLRP7* has also been linked to chromatin-level regulation of DNA methylation through interactions with additional partner proteins; in human embryonic stem cells, *NLRP7* knock-down causes aberrant CpG methylation, accelerated trophoblast differentiation and elevated hCG—phenotypes mirroring HM [52,53]. Mechanistically, it is useful to distinguish the two arms of imprint biology that *NLRP7* influences. The establishment of maternal methylation imprints occurs during oocyte growth and is catalyzed by the de novo methyltransferase DNMT3A, together with its cofactor DNMT3L [54,55], whereas the maintenance of these parent-of-origin marks through the genome-wide reprogramming of early cleavage divisions depends on DNMT1 and its accessory factors UHRF1 and DPPA3 (STELLA) [56,57,58], with imprint-specific protection additionally provided by the KRAB–zinc-finger proteins ZFP57 and ZNF445 acting through TRIM28 [59,60,61]. As a cytoplasmic, oocyte-restricted protein, NLRP7 does not methylate DNA directly; rather, it is thought to safeguard this machinery from the oocyte cytoplasm. A recent structural and functional study showed that NLRP7 binds the DNMT3A inhibitor TCL1A and sequesters it in the cytoplasm, thereby preventing TCL1A from entering the nucleus and suppressing DNMT3A-mediated de novo methylation; recurrent-mole-associated NLRP7 variants impair the NLRP7–TCL1A interaction and so release this inhibitory brake, providing a concrete molecular route from NLRP7 loss-of-function to the maternal-DMR hypomethylation that characterizes biparental HM [62]. This cytoplasmic-to-nuclear regulatory logic complements the SCMC scaffolding and chromatin-associated functions described above and helps explain why a single maternal-effect gene can produce genome-wide, placenta-biased imprinting failure. Failure of *NLRP7*-mediated imprint regulation is therefore considered the core epigenetic basis of biparental HM.

## 4. Oncogenic Mechanisms of NLRP7 in the Progression from Hydatidiform Mole to Gestational Trophoblastic Neoplasia

### 4.1. From Trophoblast Hyperproliferation to Pre-Malignant Disease

Normal trophoblast development requires a tightly regulated balance between proliferation and differentiation [63]. Loss of *NLRP7* fundamentally perturbs this balance. Recent single-nucleus multi-omics studies—integrating snRNA-seq, ATAC-seq and spatial transcriptomics—have systematically dissected trophoblast heterogeneity in CHM and demonstrated disrupted differentiation hierarchies across all three major trophoblast lineages (villous cytotrophoblasts, syncytiotrophoblasts, extravillous trophoblasts), with cell-type-specific dysregulation of imprinted-gene expression [64]. *NLRP7* deficiency therefore does not simply affect a single trophoblast subtype: it derails the entire trophoblast differentiation hierarchy, driving excessive self-renewal of stem-like villous cytotrophoblasts and impaired terminal differentiation. The resulting unrestrained proliferation provides the cellular substrate from which malignant transformation can emerge.

In vitro studies confirm a dosage-dependent role for *NLRP7* in trophoblast biology. In normal trophoblasts, moderate *NLRP7* expression promotes proliferation while restraining premature differentiation; in malignant trophoblasts, aberrantly elevated *NLRP7* expression further drives proliferation through inflammasome-independent pathways [65]. Patient-derived iPSC trophoblast-differentiation models show that *NLRP7* loss-of-function causes precocious down-regulation of pluripotency factors and premature activation of trophoblast-lineage markers in a *BMP4*-dependent manner; pharmacological BMP-pathway inhibition partially corrects excessive trophoblast differentiation in patient iPSCs, providing proof-of-concept evidence for upstream-pathway intervention [30,66,67].

### 4.2. Imprinting Disruption, Trophoblast Identity, and the HLA-C Immune-Tolerance Axis

Precise maintenance of genomic imprinting underpins normal trophoblast identity. *NLRP7* deficiency disrupts methylation of maternal DMRs and dysregulates several imprinted loci, including those governing growth and differentiation. In extravillous trophoblasts, HLA-C expression is regulated by an *ELF3*–*NLRP2*/*NLRP7* axis: *ELF3* activates *NLRP7* transcription via enhancer binding, with downstream up-regulation of HLA-C contributing to the unique semi-allogeneic immune-tolerance phenotype of trophoblasts at the maternal–fetal interface [68,69,70]. Loss of *NLRP7* disrupts this axis, with predicted consequences for both pregnancy maintenance and immune surveillance of trophoblastic neoplasia. Progesterone receptor membrane component 1 (*PGRMC1*) further modulates *NLRP7* protein levels through autophagy, and disruption of the *PGRMC1*/*NLRP7*/HLA-C axis has been linked to spontaneous preterm birth and gestational choriocarcinoma [71].

### 4.3. NLRP7 in Choriocarcinoma: A Paradoxical Pro-Tumorigenic Role

A critical feature of *NLRP7* biology is its apparently context-dependent role: maternal-effect loss-of-function drives molar disease, whereas in established choriocarcinoma cells, *NLRP7* protein is reported to be up-regulated and to behave as a tumor-supporting factor. In choriocarcinoma cell lines and in patient sera, *NLRP7* protein and circulating inflammatory cytokines have been found to be markedly elevated, and *NLRP7* has been reported to support tumor-cell proliferation, three-dimensional spheroid reorganization, and the establishment of an immunosuppressive microenvironment through inflammasome-independent pathways [26]. It should be noted that this pro-tumorigenic role of *NLRP7* in choriocarcinoma currently derives largely from a single group of related studies and a limited number of cell-line models, and it awaits independent confirmation in additional models and primary tumor cohorts. Mechanistically, *NLRP7* cooperates with βhCG, HLA-G/HLA-C and PD-L1 [72,73] to enable choriocarcinoma cells to evade maternal immune surveillance [27,68]. These findings reposition *NLRP7* as a candidate therapeutic target warranting further investigation in choriocarcinoma and emphasize that loss-of-function in the oocyte and gain-of-pathological-function in the tumor are not contradictory but reflect the gene’s dosage- and context-dependence. Several non-mutually exclusive mechanisms have been proposed to link aberrant NLRP7 activity to malignant progression. First, in choriocarcinoma cells, NLRP7 appears to act largely through inflammasome-independent signaling, sustaining proliferation and three-dimensional reorganization while elevating secretion of pro-inflammatory and pro-angiogenic cytokines that remodel the tumor microenvironment. Second, NLRP7 contributes to immune escape: by feeding into the ELF3–NLRP2/NLRP7 axis that governs HLA-C, and by cooperating with HLA-G and PD-L1, it helps trophoblastic tumor cells co-opt the physiological maternal–fetal tolerance program to evade immune surveillance. Third, the same imprinting and methylation disturbances that initiate molar disease—genome-wide loss of maternal-DMR methylation and dysregulated expression of growth-controlling imprinted loci such as CDKN1C (p57^KIP2^)—create a permissive epigenetic background in which hyperproliferative trophoblasts can accumulate further oncogenic changes. Finally, post-transcriptional control of NLRP7 abundance, for example PGRMC1-dependent autophagic turnover, modulates the PGRMC1/NLRP7/HLA-C axis implicated in choriocarcinoma, suggesting that the level rather than merely the presence of NLRP7 protein shapes tumor behavior. These mechanisms remain to be integrated and independently validated, but together they outline how a maternal-effect imprinting regulator can be repurposed as a tumor-supporting factor during the hydatidiform-mole-to-GTN transition.

### 4.4. GTN Risk in NLRP7 Mutation Carriers

Several cohort studies have reported a higher cumulative incidence of GTN in women with biallelic *NLRP7* mutations than in the sporadic-HM population. A 17-year prospective Indian study found a substantially elevated GTN rate in this group [23]; case reports and small case series have additionally documented multi-agent-chemotherapy-requiring high-risk GTN refractory to standard single-agent regimens in homozygous mutation carriers [24]. The precise relative risk should, however, be interpreted with caution because available data come predominantly from referral-center cohorts of clinically severe RHM, with attendant ascertainment bias; population-based estimates are still lacking, and the direction of this bias is not self-evident: referral enrichment may overstate the per-pregnancy GTN risk, whereas under-recognition of milder carriers who never reach a specialist center may understate the proportion of all GTN that is genetically determined. Conceptually, biallelic *NLRP7* mutation is best regarded not merely as a cause of reproductive failure but as a heritable cancer-predisposition state: it specifies a defined population in whom a pre-malignant lesion arises with near-complete penetrance and progresses to malignancy at an elevated rate. Framing the condition in these terms aligns it with other monogenic tumor-predisposition syndromes and provides an explicit rationale for genotype-informed, risk-stratified surveillance rather than uniform follow-up. Pragmatically, all patients with confirmed biallelic *NLRP7* mutations warrant rigorous, protocolized post-evacuation hCG surveillance and early oncological referral. In practical terms, and consistent with established gestational-trophoblastic-disease protocols, this entails serial quantitative serum hCG measurement until three consecutive weekly results are normal, followed by monthly determinations; given the elevated GTN risk in this population, clinicians should adopt a low threshold for extending monitoring and for early imaging if hCG plateaus or rises. Because the underlying defect is heritable and lifelong, surveillance considerations apply to every subsequent pregnancy, irrespective of the reproductive strategy chosen.

## 5. Phenotypic Spectrum of Biallelic NLRP7 Mutations

### 5.1. Complete Hydatidiform Mole as the Dominant Phenotype

Complete HM (CHM) is the most common reproductive outcome in women with biallelic *NLRP7* mutations. Crucially, the genetic signature of these moles differs fundamentally from sporadic CHM: while sporadic CHM is typically androgenetic with an entirely paternal genome, *NLRP7*-associated recurrent CHM is biparental diploid [74], containing both paternal and maternal chromosomes yet displaying CHM-like pathology because of maternal-imprinting failure [75]. This observation established that maternal-effect-gene deficiency, not parental-genome origin alone, dictates the abnormal trophoblast-proliferation phenotype. Patients with homozygous loss-of-function variants typically experience consecutive CHMs, with virtually every pregnancy resulting in molar disease and exceedingly rare spontaneous live births [76,77,78].

### 5.2. Partial Hydatidiform Mole and Spontaneous Abortion

The phenotypic spectrum is, however, not invariable. Some carriers present with partial HM (PHM) or recurrent spontaneous abortion, representing relatively milder reproductive outcomes. In family studies, PHM is more frequently observed in compound heterozygous carriers than in homozygous truncating carriers, suggesting that residual protein function may attenuate phenotypic severity [79]. Recurrent spontaneous abortion is also a significant phenotype and reflects the broad impact of *NLRP7* deficiency on oocyte function and preimplantation development. Beyond the biallelic disease that is the focus of this review, accumulating evidence indicates that women carrying a single heterozygous *NLRP7* variant may also be at increased risk of reproductive wastage, albeit through a milder and less penetrant mechanism. Heterozygous carriers have been reported with recurrent miscarriage and, in some pedigrees, molar pregnancies, and the offspring of heterozygous mothers can show multilocus imprinting disturbances—observations interpreted as an autosomal-dominant maternal-effect contribution rather than the fully penetrant recessive phenotype [80,81,82]. Consistent with this, heterozygous variants have been documented in series of women with recurrent pregnancy loss accompanied by multilocus methylation defects [49,83]. The clinical significance of an isolated heterozygous *NLRP7* variant nonetheless remains uncertain—such variants are also found in fertile controls, and penetrance is clearly incomplete—so heterozygosity is best viewed as a risk modifier whose interpretation requires caution in counseling. Incorporating heterozygous carriers makes clear that the reproductive phenotype associated with *NLRP7* spans a continuum, from isolated recurrent pregnancy loss in some heterozygotes through partial and complete molar disease in biallelic carriers.

### 5.3. Genotype–Phenotype Severity Grading

The pathogenic effect of *NLRP7* variants tracks closely with predicted protein consequence. Variants causing complete loss of protein function (nonsense, frameshift, large deletions) produce the most severe phenotype—near-universal CHM and an extremely low probability of normal autologous pregnancy. Missense variants that may preserve partial protein function are associated with relatively milder phenotypes, with occasional reports of normal pregnancies [84]. Splice-site variants occupy an intermediate position depending on their actual effect on mRNA processing. Systematic severity grading [85] has direct implications for genetic counseling and oncological surveillance [86]. Table 1 summarizes the genotype–phenotype correlations across variant categories, together with representative variants drawn from the indicated primary references.

### 5.4. Phenotypic Heterogeneity in Family Studies

Family studies reveal striking phenotypic heterogeneity, even among *NLRP7* carriers sharing identical genotypes. Three Chinese pedigrees illustrate that patients carrying identical homozygous or compound-heterozygous variants can present with phenotypes ranging from CHM through PHM to recurrent spontaneous abortion [87]. Such heterogeneity is particularly prominent in compound-heterozygous families, where sisters with identical biallelic mutations have shown markedly different clinical trajectories, implicating modifier genes, epigenetic background, parental age, and environmental factors as additional determinants of phenotypic outcome.

## 6. The Genetic Permissiveness Framework for Autologous-Oocyte Pregnancy

### 6.1. Conceptual Basis: Maternal-Effect Genes and the Zygotic-Genome-Activation Window

Maternal-effect genes encode proteins that accumulate during oogenesis and exert critical regulatory functions in the early post-fertilization embryo before ZGA. In humans, ZGA occurs at the four- to eight-cell stage [88,89,90]; until then, normal embryonic development depends entirely on the reserve and integrity of maternal factors. As a canonical maternal-effect gene, *NLRP7* protein accumulates abundantly in growing oocytes and remains active in imprint maintenance during the early embryo until the zygotic genome assumes regulatory control [91].

This temporal architecture provides the conceptual basis for genetic permissiveness for autologous-oocyte pregnancy. If residual maternal *NLRP7* protein can sustain methylation of the most critical imprinted loci until ZGA, the embryo may, in principle, traverse the early-developmental danger window and enter the phase of autonomous zygotic regulation. Once imprinting defects accumulate before ZGA, repair is exceedingly difficult, and the embryo proceeds irreversibly toward the HM phenotype. The functional threshold of maternal *NLRP7* protein and the temporal width of the imprint-maintenance window therefore jointly define the boundaries of genetic permissiveness for any given mutation.

### 6.2. Reported Live Births from Autologous Oocytes: Genotype Analysis

Live births from autologous oocytes in women with biallelic *NLRP7* mutations are exceedingly rare. A Mexican cohort study identified a regional founder [92] *NLRP7* missense variant in a population enriched for RHM and reported a small number of normal pregnancies in carriers, including spontaneous live births in patients carrying the p.L750V missense allele rather than truncating variants [29]. Although founder-effect bias and the small number of events limit the generalizability of these observations, the genotypic enrichment is consistent with the hypothesis that residual protein function—rather than complete loss—underlies rare permissive pregnancies. From a molecular perspective, comprehensive genotype–phenotype analyses of pregnancy tissues have shown that truncating variants are associated with complete loss of *CDKN1C* (p57^KIP2^) staining, absence of inner-cell-mass-derived embryonic tissue and trophoblast hyperproliferation, whereas certain missense variants show variably retained *CDKN1C* expression, detectable embryonic tissue, and relatively less aggressive trophoblast proliferation, providing direct evidence for a quantitative gradient of imprint-regulation failure [93]. Representative reported live births in biallelic carriers, together with their genotypes, conception routes and outcomes, are summarized in Table 2; the pattern reinforces two points—autologous-oocyte live births are essentially restricted to carriers of mild missense alleles, and the majority of documented successful pregnancies in this population have been achieved through oocyte donation.

### 6.3. Defining Genetic Permissiveness and Its Determinants

Across published cohorts, the overall probability of a normal pregnancy outcome in women with biallelic *NLRP7* mutations appears to be very low—reported live births from autologous oocytes remain anecdotal—underscoring the extreme constraint of genetic permissiveness in this population and the need for individualized risk assessment. We define *genetic permissiveness* as the biological probability that an oocyte from a woman carrying a specific *NLRP7* mutation retains sufficient residual molecular function to support the fertilized egg through the critical early-embryonic developmental window and achieve a normal pregnancy outcome. The term *genetic permissiveness*, as used here, is introduced in the present review as an integrative conceptual construct; it is not an established entity in the prior literature, and it is offered to organize existing observations and to generate testable hypotheses rather than to denote a validated clinical parameter. We propose three non-mutually exclusive mechanisms that may shape this permissiveness. To make the construct operational rather than purely descriptive, we suggest that variants be provisionally stratified using explicit, if still tentative, criteria that combine genetic and functional evidence. Under this scheme, a variant would be considered likely non-permissive if it is a biallelic complete loss-of-function genotype (homozygous or compound-heterozygous nonsense, frameshift, canonical splice, or whole-gene/exon deletion) predicted to undergo nonsense-mediated decay, classified as pathogenic by ACMG criteria, and—where data exist—associated with absent CDKN1C (p57^KIP2^) staining, absent embryonic tissue, and uniform multilocus maternal-DMR hypomethylation. A variant would be considered possibly permissive only if at least one allele is a missense (or hypomorphic) change with predicted partial retention of function, supported where possible by in silico concordance, by demonstrable residual activity in patient-derived iPSC trophoblast or methylation assays, and ideally by a documented family history of a non-molar pregnancy on that genotype. Genotypes that meet neither set of criteria—including most splice and rare missense variants of uncertain significance—should be classified as indeterminate and managed conservatively as non-permissive until functional data accrue. Crucially, these categories are intended as a structured framework for research-grade counseling rather than as validated thresholds: they define what evidence would need to be assembled for any given variant, and they translate directly into the counseling pathway set out in Section 6.4, in which only the possibly permissive category warrants discussion of investigational autologous-oocyte approaches, always alongside oocyte donation as the evidence-based default.

#### 6.3.1. Hypothesis 1—Residual Protein Function

Nonsense, frameshift and large-deletion mutations typically eliminate the protein outright or trigger nonsense-mediated mRNA decay, reducing residual protein to near-zero levels. Certain missense mutations affect only local protein structure, preserving partial DNA-methylation–regulatory capacity. Patient-derived iPSC models have provided supportive evidence: iPSCs carrying different *NLRP7* variant types show graded functional differences during trophoblast differentiation that correlate with clinical phenotypes, with higher residual protein function associated with a lower tendency to trophoblast hyperproliferation [30].

#### 6.3.2. Hypothesis 2—Imprint-Escape Window

Even when maternal *NLRP7* function is impaired, certain critical imprinted loci may be transiently protected by alternative maintenance mechanisms in the early post-fertilization period, creating a brief “imprint-escape window” during which the embryo can complete ZGA and assume autonomous imprint maintenance. This hypothesis is not in conflict with the irreversibility of imprinting defects once established (Section 3.3): the two statements describe opposite ends of the same threshold. The escape window posits that, in a minority of conceptuses, residual or redundant maintenance activity prevents critical defects from accumulating in the first place; once that threshold is crossed, however, repair remains effectively impossible. Genetic permissiveness therefore reflects the probability of staying below the defect threshold, not of reversing damage after it has occurred. In *NLRP7* variant-carrying miscarriage specimens, the degree of multilocus imprinting disturbance correlates with pregnancy loss, but some specimens display incomplete imprinting disruption, suggesting that imprint escape is biologically possible. The width of this window is likely modulated by mutation severity, modifier-gene background, and the oocyte microenvironment. We emphasize that the imprint-escape window remains a speculative mechanism: no study has directly demonstrated a transient, locus-specific protective activity operating in human conceptuses from biallelic NLRP7 carriers. The supporting evidence is indirect, resting on the observation that some miscarriage specimens show incomplete rather than uniform imprinting disruption [49,51]; this hypothesis is therefore offered to motivate targeted methylation-kinetics studies rather than as an established phenomenon.

#### 6.3.3. Hypothesis 3—Allelic Dosage Effects

Compound-heterozygous carriers, by definition, harbor two alleles whose mutational consequences may differ; the cumulative protein function may therefore exceed that of homozygous biallelic loss-of-function carriers. When at least one allele in a compound-heterozygous configuration is a function-retaining missense variant, residual protein output may suffice to cross the functional threshold for autologous-oocyte pregnancy. This dosage effect is indirectly supported by phenotypic-heterogeneity studies in which compound-heterozygous carriers more frequently display milder phenotypes (PHM, recurrent spontaneous abortion) than homozygous truncating carriers. This dosage model, too, should be regarded as provisional: it is inferred from genotype–phenotype associations [79,87] rather than from direct quantification of residual NLRP7 protein or methylation-maintenance activity per allele, and additive allelic contributions have not been formally demonstrated. Allele-resolved functional assays in patient-derived models will be required to test whether cumulative protein output, as opposed to other modifiers, accounts for the milder phenotypes observed in compound-heterozygous carriers.

### 6.4. Translating Permissiveness into Counseling

**Figure 1:**
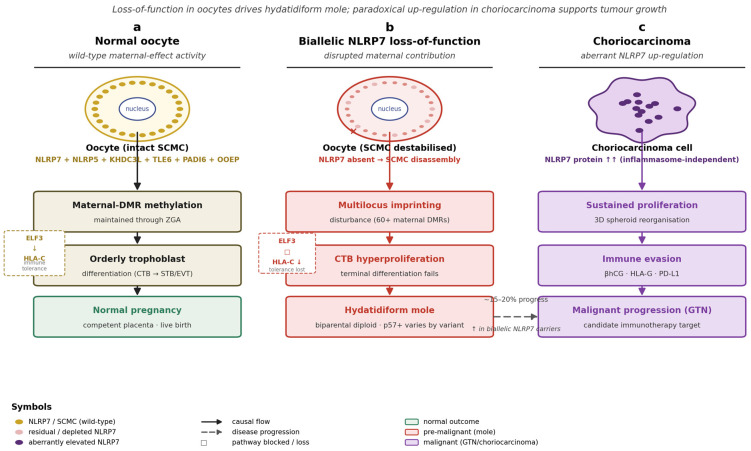
Schematic comparison of molecular pathways under normal *NLRP7* function (**a**) and biallelic *NLRP7* loss-of-function (**b**). In normal oocytes, *NLRP7* protein participates in the subcortical maternal complex (SCMC), supports maternal-DMR methylation at imprinted loci, and enables orderly trophoblast differentiation through preimplantation development. In oocytes from biallelic *NLRP7* carriers, SCMC integrity is compromised, maternal DMRs undergo widespread hypomethylation, and trophoblast differentiation is derailed: villous cytotrophoblasts hyperproliferate, terminal differentiation fails, and the conceptus develops as a hydatidiform mole. In choriocarcinoma, *NLRP7* protein is paradoxically up-regulated and supports tumor growth and immune evasion through inflammasome-independent pathways. SCMC = subcortical maternal complex; ZGA = zygotic genome activation; GTN = gestational trophoblastic neoplasia.

**Figure 2:**
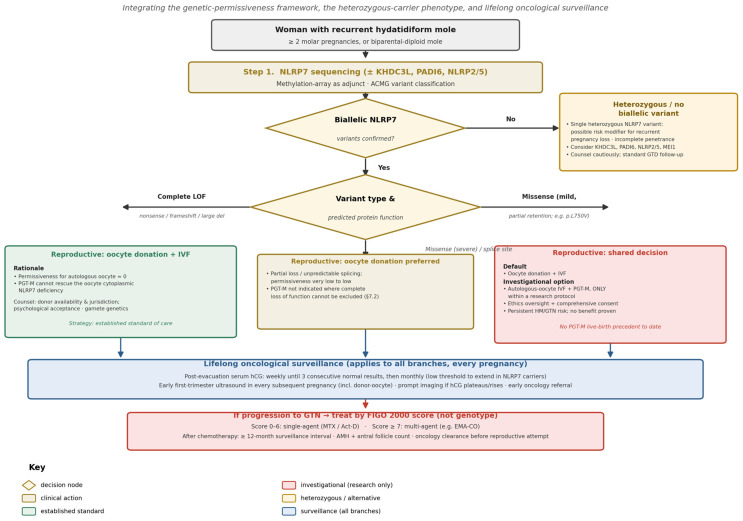
Clinical decision algorithm for reproductive management of women with biallelic *NLRP7* mutations, integrating the genetic-permissiveness framework with oncological surveillance. Following confirmation of biallelic *NLRP7* mutations, patients are stratified by variant type and predicted protein function. For complete loss-of-function variants (nonsense, frameshift, large deletions), oocyte donation with IVF is recommended as the primary strategy. For missense variants with predicted partial function retention, individualized assessment incorporating variant pathogenicity (ACMG), available functional data (iPSC-trophoblast assays, methylation studies), family history, and patient preferences guides shared decision-making, with oocyte donation as the default option and autologous-oocyte IVF with PGT-M considered only as an investigational alternative within a research protocol and under ethics oversight (no live-birth precedent reported to date). All patients require post-evacuation hCG surveillance and oncological follow-up regardless of reproductive strategy. For patients with a history of GTN and chemotherapy, additional ovarian-reserve assessment and oncological clearance precede reproductive attempts. Where biallelic variants are not confirmed, the algorithm now also accommodates women carrying a single heterozygous NLRP7 variant, in whom such a variant may act as a risk modifier for recurrent pregnancy loss with incomplete penetrance and warrants cautious counseling and consideration of other maternal-effect genes. If gestational trophoblastic neoplasia supervenes in any branch, chemotherapy intensity is determined by the FIGO 2000 prognostic score (single-agent for scores 0–6; multi-agent, e.g., EMA-CO, for scores ≥ 7) rather than by genotype. PGT-M = preimplantation genetic testing for monogenic disorders; ACMG = American College of Medical Genetics and Genomics.

For homozygous nonsense or frameshift carriers, the three mechanisms converge on a near-zero permissiveness prediction: there is no theoretical basis for residual protein function, the imprint-escape window is expected to be vanishingly narrow under complete functional loss, and allelic-dosage effects are inapplicable. Evidence strongly supports oocyte donation as the primary reproductive strategy. Conversely, for compound-heterozygous carriers in whom at least one allele is a missense variant with predicted partial function retention, individualized assessment integrating variant-pathogenicity classification (ACMG criteria), in silico prediction, available functional data (iPSC trophoblast assays, in vitro methylation studies), and detailed family reproductive history may identify cases in which cautious exploration of autologous-oocyte IVF combined with PGT-M may be considered—but, as detailed in Section 7.3, only as an investigational option within a research protocol, under comprehensive informed consent and ethics committee oversight, and with explicit acknowledgement that no live birth through PGT-M has yet been reported in biallelic *NLRP7* carriers. Figure 1 illustrates the proposed molecular pathways under normal versus mutant *NLRP7* conditions, and Figure 2 presents the clinical decision algorithm operationalizing the framework.

## 7. Assisted Reproductive Strategies and Oncological Co-Management

### 7.1. Oocyte Donation: Current Standard and Limitations

Oocyte donation remains the most evidence-based reproductive strategy for women with biallelic *NLRP7* mutations. By bypassing the carrier’s own oocyte maternal-factor deficiency, donation removes the genetic interference with imprint establishment and early embryonic development, allowing the fertilized egg to develop in a competent maternal molecular environment. Case reports confirm successful term live births in biallelic *NLRP7* mutation carriers following oocyte donation, with pregnancy courses comparable to standard assisted-reproduction patients [94].

Oocyte donation, however, has limitations. The offspring lacks a genetic connection to the birth mother, which poses psychological-acceptance barriers for some patients. Donor-oocyte availability is highly variable across jurisdictions: in mainland China, current regulations sharply limit access; some European jurisdictions prohibit anonymous donation. These regulatory differences mandate region-specific counseling. Finally, oocyte donation does not eliminate the carrier’s own ongoing GTN risk: post-evacuation oncological surveillance must continue regardless of subsequent reproductive strategy, and any future pregnancy—including donor-oocyte pregnancies—should still trigger early ultrasound for trophoblastic features.

### 7.2. PGT-M for NLRP7: Technical Possibilities and Fundamental Limits

Preimplantation genetic testing for monogenic disorders (PGT-M) provides a technical pathway for genetic screening within an autologous-oocyte IVF framework. Next-generation-sequencing-based PGT-M protocols can determine parental origin and chromosomal ploidy through copy-number-variation calling and heterozygous-SNP-based haplotyping in single-cell amplification products, identifying embryos free of pathogenic *NLRP7* variants [95]. However, PGT-M for *NLRP7* faces a fundamental—and frequently underappreciated—limitation: PGT-M screens only the embryonic nuclear genome and cannot remedy the cytoplasmic maternal-protein deficiency carried by the oocyte itself. Even an embryo with a wild-type or heterozygous nuclear genotype, derived from an oocyte lacking sufficient maternal *NLRP7* protein, will encounter imprint-maintenance failure during the pre-ZGA window. PGT-M is therefore conceptually appropriate only when residual maternal *NLRP7* protein is biologically plausible—i.e., in the missense-with-partial-retention scenario. Two further points should be made explicit to avoid overstating the evidence. First, the technical feasibility of PGT-M in RHM patients—the ability to determine parental origin and ploidy in single-cell amplification products—has been demonstrated [95], but technical feasibility is not clinical efficacy: to our knowledge, no live birth achieved through PGT-M has so far been reported in a biallelic *NLRP7* carrier. Second, the rare autologous-oocyte live births reported in carriers of mild missense variants such as p.L750V occurred as spontaneous pregnancies rather than as outcomes of PGT-M [29]; they support the existence of residual protein function but cannot be cited as evidence that PGT-M as an intervention improves outcomes. PGT-M for *NLRP7* should therefore be regarded, at present, as a research-stage strategy whose role is to be defined within prospective protocols, and not as an established alternative to oocyte donation.

### 7.3. Autologous-Oocyte IVF with PGT-M: Feasibility and Risk

Autologous-oocyte IVF combined with PGT-M should therefore be understood as an investigational strategy applicable, if at all, only to a narrowly defined subgroup—carriers of missense variants with predicted partial protein retention and, ideally, demonstrable residual function in supporting in vitro models—rather than as an established alternative to oocyte donation. For homozygous complete-loss-of-function variants, and for variants in which complete loss of function cannot be excluded, both theoretical reasoning and the available empirical evidence predict failure, and autologous-oocyte IVF with PGT-M offers no realistic clinical benefit; oocyte donation is the appropriate recommendation. For carriers of mild missense variants with retained function, the central limitation must be stated plainly: PGT-M cannot correct the oocyte’s cytoplasmic maternal-protein deficiency (Section 7.2), and no live birth achieved through PGT-M has yet been reported in this population. Where such carriers nonetheless wish to pursue autologous-oocyte use after being fully informed of these limitations, this is most appropriately undertaken within a prospective research protocol or registry rather than as routine care, and only under (i) comprehensive informed consent that explicitly addresses the persistent risk of HM and GTN and the absence of proven benefit, (ii) institutional ethics committee oversight, (iii) early first-trimester ultrasound surveillance for molar features, and (iv) protocolized post-evacuation hCG monitoring for any pregnancy that does not progress normally. Framing the option in these terms protects patient autonomy while avoiding any implication that PGT-M is an evidence-based solution.

### 7.4. Fertility Preservation and Reproductive Planning After GTN

For *NLRP7* mutation carriers who have progressed to GTN and undergone chemotherapy, fertility preservation and reproductive planning sit at the intersection of oncology and reproductive genetics. *NLRP7*-associated GTN is, like all GTN, treated according to the FIGO 2000 prognostic [96,97] scoring system rather than on the basis of genotype: patients with a score of 0–6 are classified as low-risk and receive single-agent chemotherapy (methotrexate or actinomycin-D), whereas those scoring 7 or higher are high-risk and receive multi-agent regimens such as EMA-CO, which carry greater gonadotoxicity [98,99,100]. Current evidence that biallelic *NLRP7* carriers more often fall into the high-risk, multi-agent category is limited to small case series and isolated reports of single-agent-resistant disease [24]; whether genotype independently predicts a higher FIGO score, beyond the established clinical and biochemical risk factors, has not been established and should be tested in adequately powered cohorts. Systematic data on post-chemotherapy ovarian reserve specifically in this population are extremely limited, and the inherent oocyte-quality impairment caused by *NLRP7* deficiency further complicates fertility-preservation strategies—oocyte cryopreservation in carriers of complete loss-of-function variants is, in particular, of debatable value because the cryopreserved oocytes will themselves carry the maternal-factor deficiency. Pre-chemotherapy fertility-preservation counseling should therefore be honest about these constraints. Post-treatment, the standard oncological recommendation is a minimum 12-month surveillance interval before any subsequent pregnancy attempt, with comprehensive ovarian-reserve assessment (anti-Müllerian hormone, antral follicle count) before reproductive planning. Oocyte donation remains the default strategy in this context. If a patient strongly prefers autologous-oocyte use, variant-type-dependent PGT-M and stringent pregnancy monitoring are warranted, with explicit disclosure of the limited evidence base. Multicenter prospective registries are urgently needed to optimize fertility-preservation protocols specifically for this population [12].

## 8. Strengths, Limitations and Future Directions

This review integrates oncological, genetic, epigenetic and reproductive-medicine perspectives on a rare but high-stakes condition, drawing on the most recent multi-omics, structural and iPSC-based functional studies. Its principal strength is the explicit linkage of cancer-surveillance considerations to reproductive decision-making, an integration that has been largely absent from prior reviews focused solely on either tumor biology or fertility. Two further conceptual contributions are, to our knowledge, novel: the explicit reframing of biallelic *NLRP7* mutation as a heritable cancer-predisposition state with near-complete penetrance of a pre-malignant lesion, and the genetic-permissiveness construct, which converts an unresolved binary clinical question into a graded, variant-level and empirically testable prediction.

Several limitations should be acknowledged. First, the extreme rarity of biallelic *NLRP7* mutations means that the evidence base consists predominantly of case reports and small case series; estimates of GTN risk, normal-pregnancy probability, and the protective effect of specific missense variants are therefore imprecise. Second, the genetic-permissiveness framework is currently supported chiefly by indirect, mechanism-based reasoning together with a small number of observational reports; prospective validation in multicenter cohorts is essential. Third, our knowledge of modifier genes, epigenetic background and environmental cofactors that contribute to the marked phenotypic heterogeneity remains incomplete. Fourth, this is a narrative—not a systematic—review, and despite our priority weighting toward larger and functionally validated studies, selection bias cannot be excluded. Several further clinical caveats deserve emphasis. First, successful pregnancies with autologous oocytes in biallelic carriers are extraordinarily rare and have been reported almost exclusively as spontaneous conceptions in carriers of particular missense alleles; no live birth achieved through autologous-oocyte IVF with PGT-M has been documented, so the apparent permissiveness of “mild” variants rests on a very small number of anecdotal events that are highly vulnerable to publication and founder-effect bias. Second, because biallelic NLRP7 disease is so uncommon, almost all genotype–phenotype and outcome data come from small single-family or single-center series, precluding reliable estimates of penetrance, GTN risk, or the probability of a normal pregnancy for any individual variant. Third, the functional consequence of the majority of reported variants—particularly missense and splice changes—has not been experimentally validated; iPSC-trophoblast and methylation assays exist for only a handful of variants, so most permissiveness predictions remain as in silico inferences. Finally, long-term maternal outcomes (including cumulative oncological risk across successive pregnancies) and the health and imprinting status of the resulting offspring are essentially unstudied, and these must be regarded as open questions when counseling patients.

Future investigation should pursue five priorities: (i) Integration of cryo-EM-derived SCMC structural information with iPSC-trophoblast-differentiation assays to allow structure-function-based prediction of permissiveness for individual variants. (ii) Single-cell multi-omics dissection of trophoblastic neoplasia in *NLRP7*-mutant versus wild-type backgrounds, focused on the immune-microenvironment differences underlying GTN progression—an area in which *NLRP7* has been implicated as a candidate immunotherapeutic target through PD-L1, HLA-G/HLA-C and βhCG axes [26,27,68,71]. (iii) Establishment of multicenter prospective registries for *NLRP7* mutation carriers to accumulate genotype-stratified reproductive and oncological outcome data; such registries, ideally linked to population-based molar-pregnancy datasets, are also the only realistic route to unbiased estimates of the genetically determined fraction of RHM and of the absolute, population-level GTN risk that currently cannot be derived from referral-center series. (iv) Standardized genetic-counseling protocols incorporating variant-level functional data, GTN-risk stratification, and patient preferences to support shared decision-making. (v) Evaluation of emerging minimally invasive monitoring tools—including circulating tumor DNA and methylation-based liquid-biopsy assays [101]—as potential adjuncts to hCG for the early detection of malignant progression in this high-risk population, alongside trophoblast-organoid models [102,103] as a complementary functional platform for variant interpretation. Further mechanistic clarification of how loss-of-function in oocytes and gain-of-pathological-function in trophoblastic tumors are linked at the level of dosage and chromatin context remains a central conceptual gap.

## 9. Conclusions

*NLRP7* sits at the intersection of maternal-effect biology and trophoblastic oncology: its loss-of-function in oocytes drives recurrent hydatidiform mole, while its paradoxical up-regulation in choriocarcinoma supports tumor growth and immune evasion. Viewed through an epidemiological lens, biallelic mutation defines a heritable cancer-predisposition state that warrants genotype-informed oncological surveillance rather than uniform follow-up. Across reported cohorts, genetic permissiveness for autologous-oocyte pregnancy in women with biallelic *NLRP7* mutations is mutation-type dependent. For complete loss-of-function variants, oocyte donation combined with IVF remains the preferred reproductive strategy. For missense variants with partial protein retention, autologous-oocyte IVF combined with PGT-M remains, at present, an investigational option without a proven live-birth precedent, to be explored only within research protocols under comprehensive informed consent, ethics oversight, and close clinical monitoring. As iPSC-based functional assays, single-cell multi-omics, methylation-array diagnostics, and PGT-M technologies continue to mature, and as multicenter prospective data accumulate, individualized fertility-risk and cancer-risk assessment for this population will become increasingly precise—ultimately supporting truly evidence-based, integrated reproductive and oncological decision-making for each patient’s unique genotype.

## Figures and Tables

**Table 1:** Genotype–phenotype severity grading for biallelic *NLRP7* variants. Representative variants are illustrative examples reported in the indicated primary references and are not intended to be exhaustive.

Variant Type	Representative Variant (Ref.)	Predicted Protein Impact	Typical Pregnancy Outcome	GTN Risk	Autologous Oocyte Feasibility	Suggested Reproductive Strategy
Nonsense	p.Y206* [76]	Complete loss/NMD	Recurrent CHM	High	Extremely low	Oocyte donation + IVF
Frameshift	p.R458Sfs*69 [86]	Complete loss/NMD	Recurrent CHM	High	Extremely low	Oocyte donation + IVF
Large deletion	Exon 2–6 del [84]	Complete loss	Recurrent CHM	High	Extremely low	Oocyte donation + IVF
Splice site	c.2982-2A>G [86]	Variable; partial loss	CHM/PHM	Moderate	Very low to low	Oocyte donation + IVF; PGT-M not indicated where complete loss of function cannot be excluded
Missense (severe)	p.D722G [86]	Partial loss	CHM/PHM	Moderate	Low	Individualized counseling; oocyte donation preferred
Missense (mild)	p.L750V [29]	Partial retention	PHM/SA; rare live birth	Low–moderate	Limited possibility	PGT-M may be explored only within a research protocol and under informed consent (no successful PGT-M precedent reported to date)

CHM = complete hydatidiform mole; PHM = partial hydatidiform mole; SA = spontaneous abortion; GTN = gestational trophoblastic neoplasia; PGT-M = preimplantation genetic testing for monogenic disorders; NMD = nonsense-mediated mRNA decay; IVF = invitro fertilisation. Severity grading is based on predicted protein-function impact and reported reproductive outcomes; specific recommendations should always be individualized. The GTN-risk and autologous-oocyte-feasibility columns denote relative, inferential gradings extrapolated from variant-level functional consequence and the limited available outcome data rather than from direct risk estimates; they are intended to support counseling and have not been validated in prospective cohorts. Regarding the “suggested reproductive strategy” column, PGT-M for *NLRP7* is conceptually applicable only to variants with predicted partial protein retention (see Section 7.2); it is listed solely for the mild-missense category and there, to date, no live birth achieved through PGT-M has been reported in biallelic *NLRP7* carriers—the rare autologous-oocyte live births cited for this category arose from spontaneous, non-PGT-M pregnancies. PGT-M is therefore a research-stage option rather than an evidence-based intervention.

**Table 2:** Representative reported live births in women carrying biallelic *NLRP7* variants, by genotype, conception route and outcome. Cases are illustrative examples from the cited primary reports and are not exhaustive; genotypes are summarized as reported. Spontaneous (autologous-oocyte) live births are confined to carriers of mild missense alleles, whereas most documented live births in this population followed oocyte donation.

Report (Ref.)	Genotype (NLRP7)	Variant Class	Conception Route	Reproductive History	Pregnancy Outcome
Aguinaga et al. 2021 [29]	p.L750V (biallelic, founder)	Missense (partial function)	Autologous (spontaneous)	Multiple prior molar pregnancies	Spontaneous live birth(s)
Aguinaga et al. 2021 [29]	Biallelic NLRP7 variants	Mixed	Donor oocyte (IVF)	Recurrent molar pregnancies	Healthy live births
Akoury et al. 2015 [21]	Biallelic, incl. mild missense	Missense (mild)	Autologous (spontaneous)	Recurrent hydatidiform mole	Rare spontaneous live birth
Akoury et al. 2015 [21]	Biallelic NLRP7 mutations	Loss-of-function/mixed	Donor oocyte (IVF)	Recurrent hydatidiform mole	Live births from donated ova
Cozette et al. 2020 [94]	Biallelic NLRP7 mutations	Loss-of-function	Donor oocyte (IVF)	Recurrent molar pregnancies	Term live birth

## Data Availability

No new data were generated for this review. All referenced data are available in the original publications cited herein.

## Abbreviations

AMH	anti-Müllerian hormone
βhCG	β-human chorionic gonadotropin
ATAC-seq	assay for transposase-accessible chromatin sequencing
CHM	complete hydatidiform mole
DMR	differentially methylated region
GTD	gestational trophoblastic disease
GTN	gestational trophoblastic neoplasia
HLA	human leukocyte antigen
HM	hydatidiform mole
ICM	inner cell mass
iPSC	induced pluripotent stem cell
LRR	leucine-rich repeat
NACHT	NAIP/CIITA/HET-E/TP1 domain
NMD	nonsense-mediated mRNA decay
PD-L1	programmed death-ligand 1
PGT-M	preimplantation genetic testing for monogenic disorders
PHM	partial hydatidiform mole
PYD	pyrin domain
RHM	recurrent hydatidiform mole
SA	spontaneous abortion
SCMC	subcortical maternal complex
snRNA-seq	single-nucleus RNA sequencing
ZGA	zygotic genome activation
